# “I Am Going to Kill Myself”: A Self-Reflective Case Study Exploring the Suicidal Language Used in Day-to-Day Life and How It Affects Those Who Have Experienced Mental Health Difficulties

**DOI:** 10.1192/bjo.2025.10729

**Published:** 2025-06-20

**Authors:** Laura Gill

**Affiliations:** Edinburgh University, Edinburgh, United Kingdom

## Abstract

**Aims:** Some language which is now used day-to-day appears to be built upon the foundations of mental illness, such as “I am going to kill myself” or “I’m so OCD about things”. However, these phrases seem to be brushed past in so many social situations. Two years ago, I attempted suicide during a particularly difficult period of my life. My experiences have made me more perceptive of this language heard in everyday conversations. This case study aims to spark early discussions around why this language has become so common and the impact that this has on the identity and spiritual well-being of individuals affected by mental illness.

**Methods:** This is an individual self-reflective case study of my own lived experiences. It embraces reflexivity as a method to understand one’s own thoughts both past and present. During the depths of my depression, hearing others use this language made me believe that suicidal thoughts were a normal part of being human, delaying my pursuit of any mental health support. Conversely, after my suicide attempt, such language left me feeling isolated, triggering vivid flashbacks. My journey to recovery has frequently been overtaken by thoughts that my past mental health is what solely defines me as a person.

**Results:** This case study proposes that the increasing use of statements like ‘I am going to kill myself’ reflects a deeper societal need for connection to others, yet it also diminishes autonomy over when and how individuals process their own mental and spiritual experiences. Overcoming mental illness is a difficult battle, often shaking one’s sense of purpose in life. Reflecting on these experiences is essential for growth, but our society must help create a mindful and respectful space for this to happen.

**Conclusion:** A single case study goes a long way in proving that at least one individual has been affected by the use of mental health phrases by others. Whether that is the case for every person who has been mentally unwell at some point in their life or not, it is important to raise awareness of the negative setbacks it can have for vulnerable individuals. Further research should aim to recruit more participants affected by mental illness, gathering more evidence to either support or challenge the findings of this case study.

